# Extramedullary hematopoiesis within cystic renal cell carcinoma with oncocytic and chromophobe cell types: A case report

**DOI:** 10.3892/ol.2014.1801

**Published:** 2014-01-15

**Authors:** BETUL CELIK, TANGUL BULUT, MURAT SEDELE, CEM SEZER, VOLKAN KARAKUS

**Affiliations:** 1 Department of Pathology, Antalya Training Hospital, Antalya 07040, Turkey; 2 Department of Hematology, Antalya Training Hospital, Antalya 07040, Turkey

**Keywords:** extramedullary hematopoiesis, renal cell carcinoma, chromophobe, oncocyte

## Abstract

Extramedullary hematopoiesis (EMH) is a phenomenon in which hematopoietic cells are found in sites other than the bone marrow. It is usually observed in the liver and spleen but may occasionally be found within solid tumors. The current case report presents a 69-year-old female patient who presented with a renal cyst. Histopathological examination following surgical removal of the cyst revealed a lining of oncocytic- and chromophobe-type cells with capsular invasion and a mass forming EMH with evident bone trabeculae within the cyst wall. Circulating hematopoietic stem cells in the blood and their colonization within tissues is discussed in the present case report, emphasizing certain types of renal cell carcinoma.

## Introduction

Extramedullary hematopoiesis (EMH) is the formation of blood cells outside the bone marrow (BM). It may also be referred to as myeloid metaplasia and is usually observed as secondary to BM insufficiency (1). The most common location is in the liver and spleen, but it may be observed in other tissues as well as in tumors (2,3). Among the tumors, EMH has been reported in renal oncocytoma and clear cell type renal carcinoma (RCC) (4).

Despite being widely considered as an epiphenomenon, recent molecular evidence for EMH points to stem cells and the microenvironment, i.e. colonization within the liver, spleen and RCC, as a niche is unlikely to be indiscriminate. Historically, hematopoietic stem cells (HSCs) are found in the BM as well as in circulatory system. When BM insufficiency occurs, circulatory HSCs home toward the liver and spleen. However, there must be a possible crosstalk between hematopoietic and osteogenic cells, as well as a microenvironment created by certain tumors. It was found that liver sinusoidal cells had a chemokine receptor that plays important roles in the homing of HSCs (5). It was also shown that erythropoietin (EPO), which is secreted from the kidney has receptors that are present outside of hematopoietic tissues and acute bleeding may trigger bone formation as well as hematopoiesis (6) Written informed consent was obtained from the patient.

## Case report

**Figure 1:**
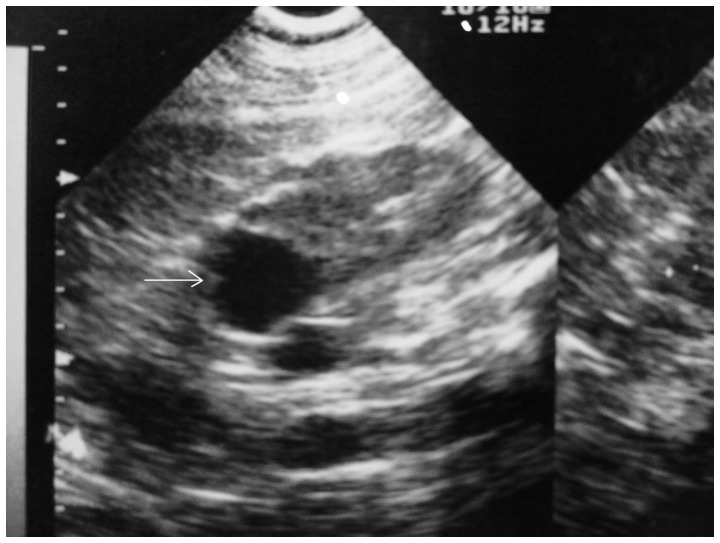
Ultrasonography of the cyst four years previously.

**Figure 2:**
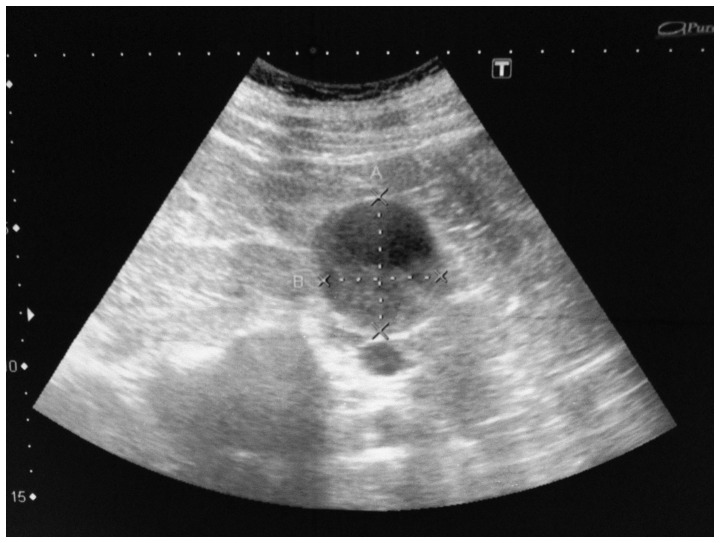
Ultrasonography of the cyst prior to surgery.

**Figure 3:**
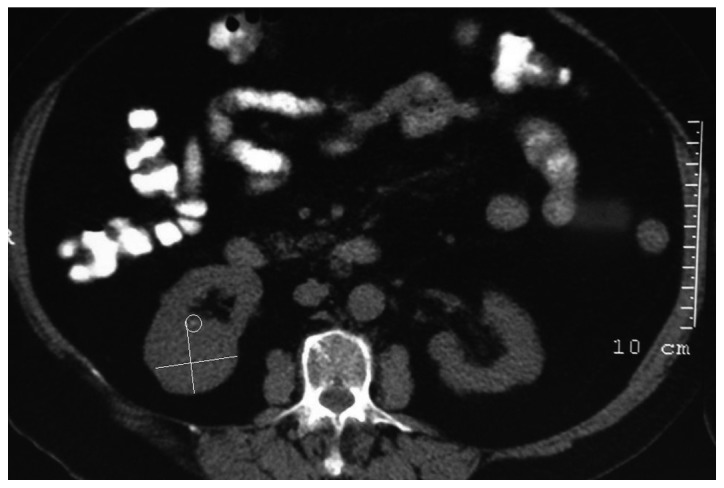
Computerized tomographic appearance of the cyst prior to surgery. Punctate calcification consistent with extramedullary hematopoiesis is circled.

**Figure 4:**
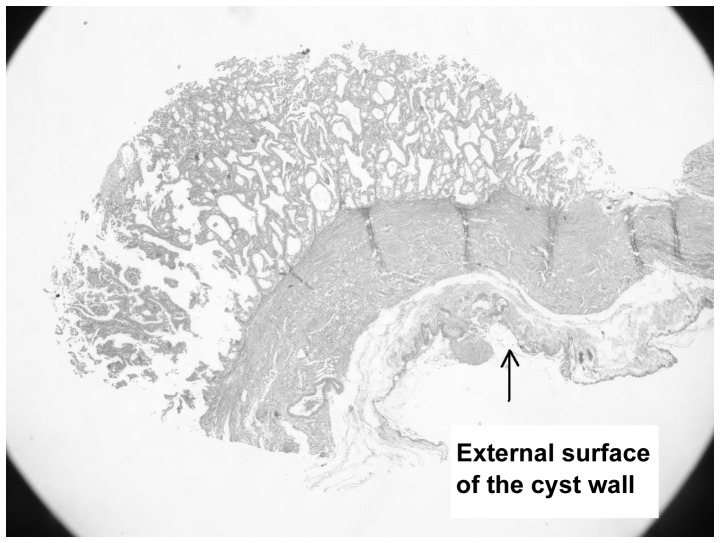
Fibrous cyst wall demonstrating the tumor focus (hematoxylin and eosin; magnification, ×2).

**Figure 5:**
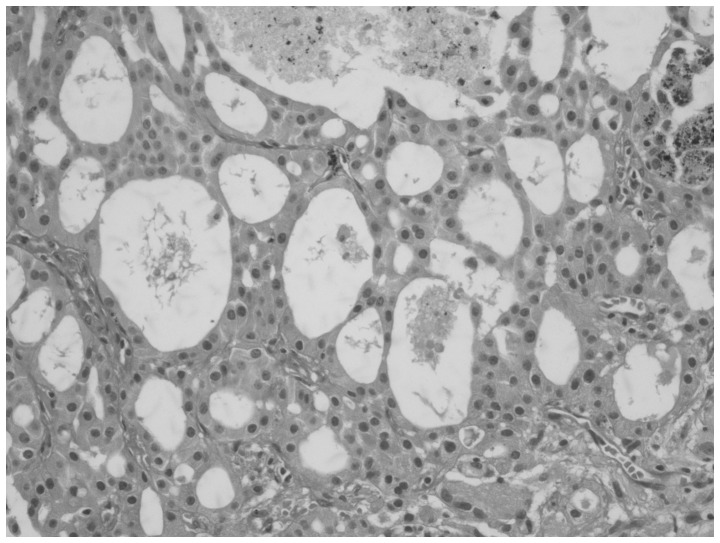
In spite of the clear cell type, in which the cytoplasm appears empty (not stained) due to glycogen content, the cell cytoplasm in this tumor contains mitochondria, which are stained by H&E (grey) (magnification, ×20).

**Figure 6:**
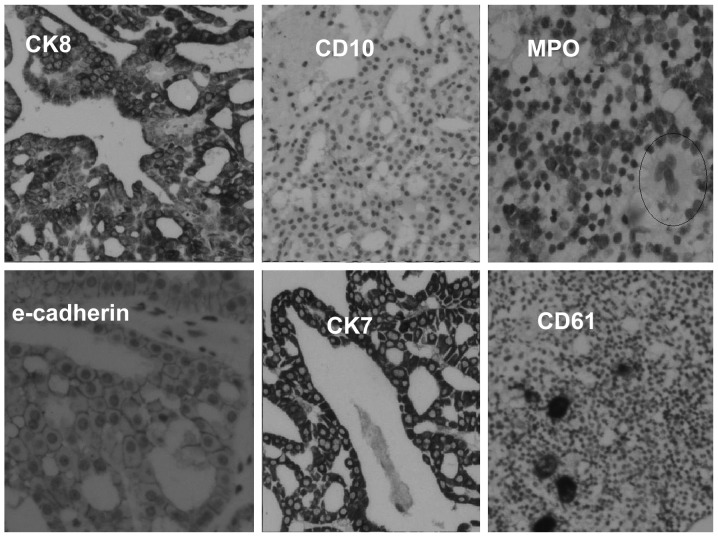
Different immunohistochemical expression of the tumor cells and immunohistological characteristics of the focus (right side). Cells negative for MPO were identified as erythroblasts. Circle indicates megakaryocytes [diffuse cytoplasmic staining of CK7 and CK8 at magnification ×10; membranous (honey-comb pattern) staining of E-cadherin at magnification ×20; negative staining of CD10 at magnification ×10; MPO, magnification ×20; CD61, magnification ×10]. MPO, myeloperoxidase.

**Figure 7:**
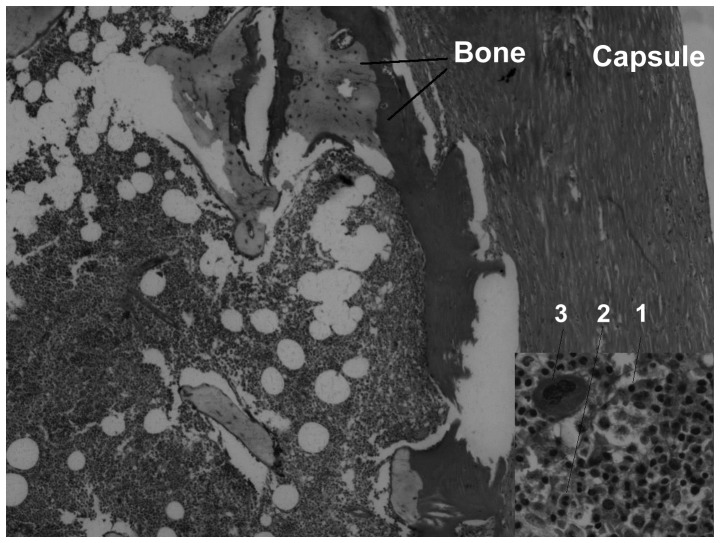
Bone trabeculae and cyst wall (H&E, ×4). The inset shows the following bone marrow cells: 1, erythroblasts; 2, myeloid series; and 3, megakaryocytes (H&E; magnification, ×20). H&E, hematoxylin and eosin.

The current report presents a case of a 69-year-old female who was incidentally found to have a renal cyst in the right kidney. The patient’s past medical history was unremarkable with the exception of a complaint of right back pain in April 2008. Further investigation revealed a right renal cortical cyst, 29 mm in diameter, by ultrasonography (USG) (Fig. 1). Later admission to the Antalya Ataturk Hospital (Antalya, Turkey) in April 2012 for the same complaint revealed an increase in the size of the right renal cortical cyst to 46×40 mm by USG (Fig. 2). The cyst was more complex and, in the base of the mass, a semilunar hyper echoic region was identified that may belong to the solid component of the tumor lesion. Abdominal tomography showed a semi-solid, exophytic situated cyst, averaging 40×35 mm in size, located at the posterior aspect of the inferior pole of the right kidney (Fig. 3). Upon admission to the hospital, the patient’s hemoglobin (Hgb) level was 13 g/dl, white blood cell (WBC) count was 6.16×10^3^/mm^3^, red blood cell (RBC) count was 4.36×10^3^/mm^3^, hematocrit (HCT) result was 39.7% and platelet (PLT) count was 244×10^3^/mm^3^ (Table I). Biochemical investigations were within normal limits. During surgery, the surgeon also identified a 5-mm mass on the cyst wall and cystectomy was performed. Histopathologically, the outer aspect of the cyst revealed atrophic tubules and sclerotic glomeruli consistent with the intraparenchymal location of the cyst (Fig. 4). The epithelial lining of the cyst consisted of cells with monotonous nuclei and oncocytic cytoplasm (Fig. 5). Tumor cells were negative for clear cell type RCC markers, CD10 and vimentin, and positive for chromophobe cell type RCC markers, E-cadherin and CK7, as well as oncocytic cell markers, CK8 and CK18 (Fig. 6). An examination of the mural nodule revealed bone trabeculae with evident BM elements (Fig. 7), which were positive for the erythroblast (glycophorin), myeloid (myeloperoxidase) and megakaryocytic (CD61) markers (Fig. 6). The histopathological appearance of the mass along with the immunohistological observations were similar to EMH.

BM biopsy was planned postoperatively but the patient did not agree to this diagnostic procedure. At the fourth postoperative month, the patient’s Hgb level was 12.1 g/dl, WBC count was 6.3×10^3^/mm^3^, RBC count was 4.1×10^3^/mm^3^, HCT result was 37.5% and PLT count was 199×10^3^/mm^3^. The Janus kinase 2 mutations and reciprocal translocation between chromosome 9 and 22, t(9;22), were not detected in the peripheral blood sample. However, they are commonly found in polycythemia vera and chronic myelogenous leukemia, respectively (7). EPO was 17,900 mU/ml in the third postoperative month. Computed tomography did not detect any residual tumor postoperatively.

## Discussion

EMH is a condition defined as the appearance of hematopoietic elements outside of the BM. It is associated with various hematological diseases, mostly chronic myeloproliferative diseases (1). The most common sites for EMH are the liver and spleen (2). However it has been previously reported in the majority of organs. EMH is usually found microscopically, but may present as a mass-forming lesion (8). The most common locations for mass-forming lesions are the paravertebral and intrathoracic spaces (9). Occasionally, these masses reach ≤8 cm in diameter, but even with this size, bone trabeculae has not previously been reported within the mass.

With the exception of the liver and spleen, EMH may affect the thoracic spinal region, which is rarely observed, and <10 cases of EMH have previously been reported in association with RCC or as occurring in the kidney (10). A number of EMHs are incidentally found and are not associated with hematological diseases. Although polycythemia is a common peripheral blood observation in RCC patients, the exact pathogenesis of EMH within RCC is not known; it has been previously reported that 74% of RCCs show EPO immunohistochemically. EPO is a hormone secreted by the kidney and fetal liver that stimulates RBC production from the BM. Expression of EPO within the tumor tissue of RCC is more frequent in clear cell type RCC (11) and has rarely been reported in oncocytomas (4). Expression of EPO has never been reported in cystic RCC or chromophobe cell type RCC, as in the present case.

Hematopoiesis begins in the yolk sac and then takes place transitorily in the liver (12,13). In adults, HSCs are primarily seen in the BM, but also in the circulatory system. Under certain circumstances, including myelofibrosis, circulating HSCs from the peripheral blood filter into the tissues. Colonization of filtered HSCs are considered to reside in specific niches, which are specialized microenvironments, such as osteoblastic cells, vascular endothelial cells, liver sinusoidal cells and reticular cells (14–16). With the collaboration of several other molecular findings (17) fetal hematopoiesis as well as EMH occurs mainly in the liver (5).

Based on aforementioned findings, it is not possible to consider EMH, which is observed in RCC or other tissues as an incidental finding. By contrast, it is possible to reflect on the association between stem cell niches and the tissues where EMH colonizes (microenvironments), i.e. certain tissues may signal HSCs. Focus of EMH is frequently observed in hepatoblastomas and, similar to RCC, EPO has been previously detected in the tumor tissues of 11 out of 15 hepatoblastomas, i.e. tissues rich in EPO, as in hepatoblastoma. In addition, RCC may signal HSCs to colonize in these tissues. EMH is also observed within the breast following therapy with granulocyte colony-stimulating factor (G-CSF) for breast cancer (18). G-CSF is a growth factor that stimulates BM to produce granulocytes and, subsequently, stem cells stimulate the BM to release the granulocytes into the blood. Breast cancer patients are treated with G-CSF for chemotherapy-related BM suppression. Since adipose tissue contains various types of adult stem cells as well as HSCs (19), the elevated levels of HSCs within the blood of the present patient may have preferentially migrated to the adipose breast tissue.

In adults, HSCs are primarily observed in the BM, but also in the blood. Notably, HSCs are the only immature cells that pass through the BM. Under certain circumstances, i.e. myelofibrosis, circulating HSCs from the peripheral blood infiltrate into the tissues. Colonization of infiltrated HSCs are considered to reside in specific niches, which are specialized microenvironments, including osteoblastic, vascular endothelial and reticular cells (16). A previous study found that the colonization of HSCs to the BM was mainly regulated by a factor named stromal-derived factor-1 (SDF1). In an additional study, SDF1 and its receptor were found to be expressed in liver sinusoidal endothelial cells (5). These two studies explain why fetal hematopoiesis occurs mainly in the liver. Based on these molecular observations and the present case report, we hypothesized that EPO produced by RCC cells raises the levels of circulating HSCs and the vascular endothelium-rich RCC acts as a niche that allows circulating HSCs to colonize within the tumor and initiate BM formation.

Bone trabecula has never been reported in RCC or cystic RCC. The precise pathway of bone trabecula in the EMH focus of the current study remains unknown. Bone trabecula has never been reported in hepatoblastomas; however, similar bone trabecula associated with EMH has been observed in the endometrium and thyroid gland (3,20). Hematological disorders have not been observed in these cases. Dystrophic calcification is a common finding in nodular goiter specimens and the presence of bone in the endometrial samples has been attributed to metaplasia following abortion and local osteogenic factors (3,20). Once bone trabecula is formed, it may serve as a niche for EMH, which has been observed in these patients. Since hypercalcemia is the most common paraneoplastic complication of RCC, bone trabecula observed in RCC may be attributed to hypercalcemia, but the current patient was normocalcemic and normocytic, pre- and postoperatively.

Although EMH is observed secondary to BM insufficiency, hematopoietic focus is observed without underlying BM insufficiency as a consequence of the presence of factors excreted by tumor cells. Hypercalcemia as well as polycythemia are frequently observed in RCC patients and EPO produced by tumor cells raises the level of blood stem cells that preferentially colonize within highly vascularized RCC. Clear cell type RCC, particularly, but also chromophobe and oncocytic cell type RCC, exhibit biological properties that result in EMH focus.

## Figures and Tables

**Table I:** Biochemical values at different time points.

Values	March 2001	May 2012	July 2012	September 2012	January 2013
WBC, mm^3^	6.82	6.16	6.3	6.3	6.6
RBC, mm^3^	4.37	4.36	4.2	4.1	4.4
Hgb, g/dl	12.9	13	12.1	12.1	12.7
HCT, %	40.6	39.7	38.6	37.5	39.9
PLT, mm^3^	228	244	193	199	202
Blood calcium, mg/dl	9.9	ND	9.5	9.6	9.9
EPO, mU/ml				17,900	
JAK-2				Negative	
t(9;22)				Negative	

WBC, white blood cell; RBC, red blood cell; Hgb, hemoglobin; HCT, hematocrit; PLT, platelet; EPO, erythropoietin; JAK-2, Janus kinase 2 mutations for myeloproliferative disorders; t(9;22), reciprocal translocation between chromosome 9 and 22; ND, no data.
